# Ontology based mining of pathogen–disease associations from literature

**DOI:** 10.1186/s13326-019-0208-2

**Published:** 2019-09-18

**Authors:** Şenay Kafkas, Robert Hoehndorf

**Affiliations:** 1 Computational Bioscience Research Center, King Abdullah University of Science and Technology, Thuwal, 23955-6900 Saudi Arabia; 2 Computer, Electrical and Mathematical Sciences and Engineering Division, King Abdullah University of Science and Technology, Thuwal, 23955-6900 Saudi Arabia

**Keywords:** Text mining, Relationship extraction, Pathogen–disease association, Pathogen, Infectious disease

## Abstract

**Background:**

Infectious diseases claim millions of lives especially in the developing countries each year. Identification of causative pathogens accurately and rapidly plays a key role in the success of treatment. To support infectious disease research and mechanisms of infection, there is a need for an open resource on pathogen–disease associations that can be utilized in computational studies. A large number of pathogen–disease associations is available from the literature in unstructured form and we need automated methods to extract the data.

**Results:**

We developed a text mining system designed for extracting pathogen–disease relations from literature. Our approach utilizes background knowledge from an ontology and statistical methods for extracting associations between pathogens and diseases. In total, we extracted a total of 3420 pathogen–disease associations from literature. We integrated our literature-derived associations into a database which links pathogens to their phenotypes for supporting infectious disease research.

**Conclusions:**

To the best of our knowledge, we present the first study focusing on extracting pathogen–disease associations from publications. We believe the text mined data can be utilized as a valuable resource for infectious disease research. All the data is publicly available from https://github.com/bio-ontology-research-group/padimi and through a public SPARQL endpoint from http://patho.phenomebrowser.net/.

## Background

Each year, millions of people die due to infectious diseases. The World Health Organisation (WHO)[1] reported that 11̇ million deaths were due to HIV/AIDS in 2015 alone. Infectious diseases cause devastating results not only on global public health but also on the countries’ economies. Developing countries, especially the ones in Africa, are the most affected by infectious diseases.

Several scientific resources have been developed to support infectious disease research. A large number of these resources focus on host–pathogen interactions [2, 3] as well as particular mechanisms of drug resistance [4]. Additionally, there are several resources that broadly characterize different aspects of diseases [5]. However, relatively little structured information is available about the relationships between pathogens and disease, information that is also needed to support infectious disease research. For example, pathogen–disease relations (and the resulting relations between pathogens and phenotypes elicited in their hosts) provide complementary information to molecular approaches to discover host–pathogen interactions [6]. More generally, however, while there is often a direct correspondence between an infectious disease and a type of pathogen, the relation between disease and the pathogen causing it needs to be available in a structured format to allow automatic processing and linking of phenotypes (i.e., disease) to the molecular mechanisms (i.e., the pathogens and their molecular interactions). Such information is further useful as some diseases can be caused by multiple types of pathogens, and the same pathogen may cause different types of diseases (e.g., depending on the anatomical site of infection).

Currently, pathogen–disease associations are mainly covered in structured format by proprietary databases such as the Kyoto Encyclopedia of Genes and Genomes (KEGG) [7]; KEGG’s DISEASE database contains a detailed classification of infectious diseases and links them to the taxon or the taxa that are known to cause the disease. For example, KEGG links the disease Tuberculosis (H00342) to two taxa: *Mycobacterium tuberculosis* and *Mycobacterium canettii*. Pathogen–disease associations are also described in the biomedical literature and public resources such as Wikipedia [8], or in the Human Disease Ontology [5] in natural language form. Automated methods are needed to extract these associations from natural language.

Here, we further developed and evaluated a text mining system for extracting pathogen–disease associations from literature [9]. While most of the existing text mining studies related to infectious disease focus on extracting host–pathogen interactions from text [10, 11] and archiving this data [2, 3], to the best of our knowledge, we present the first text mining system which focuses on extracting pathogen–disease associations. Our literature-extracted associations are available for download from https://github.com/bio-ontology-research-group/padimi and are included in PathoPhenoDB [12] and accessible through a public SPARQL endpoint at http://patho.phenomebrowser.net/.

## Materials & methods

### Ontologies and resources used

We used the latest archived version of the Open Access full text articles subset of PubMed Central (http://europepmc.org/ftp/archive/v.2017.12/, containing approximately 1.8 million articles) from the Europe PMC database [13]. We used the NCBI Taxonomy [14] (downloaded on 22-08-2017) and the Human Disease Ontology (DO) [5] (February 2018 release) to provide the vocabulary to identify pathogen and infectious disease mentions in text. We selected these two comprehensive OBO ontologies due to the fact that our method utilizes ontology structure to propagate information in relation extraction as well as interoperablity reasons. Furthermore, in a relevant study [15], we link pathogens to disease phenotypes in support of infectious disease research by utilizing the mappings from DO to phenotpes. We generated two dictionaries from the labels and synonyms in the two ontologies and refined them before applying text mining. In the refinement process, we filtered out terms which have less than three characters and terms that are ambiguous with common English words (e.g., “Arabia” as a pathogen name). We extracted the taxon labels and synonyms belonging to all fungi, viruses, bacteria, worms, insects, and protozoa from the NCBI Taxonomy to form our pathogen dictionary. The final pathogen and disease dictionaries cover a total of 1,519,235 labels and synonyms belonging to 1,250,373 distinct pathogen taxa and 1380 labels and synonyms belonging to 438 distinct infectious diseases.

### Pathogen and disease class recognition

A class is an entity in an ontology that characterizes a category of things with particular characteristics. Classes usually have a set of terms attached as labels or synonyms [16]. We used the Whatizit text mining workflow [17] to annotate pathogen and disease classes in text with the two dictionaries for diseases and pathogens. Because disease name abbreviations can be ambiguous with some other names (e.g., ALS is an abbreviation both for “Amyotrophic Lateral Sclerosis” and “Advanced Life Support”), we used a disease abbreviation filter for screening out the non-disease abbreviations that could be introduced during the annotation process [18]. Briefly, this filter operates based on rules utilizing heuristic information. First, it identifies abbreviations and their long forms in text by using regular expressions. Second, it utilizes several rules to decide whether to keep the abbreviation annotated as a disease name or filter out. The rules cover keeping the abbreviation either if any of its long forms from DO exists in the document or its long form contains a keyword such as “disease”, “disorder”, “syndrome”, “defect”, etcṫhat describes a disease name.

### Pathogen–Disease association extraction

Our association extraction method is based on identification of pathogen–disease co-occurrences at the sentence level and applying a filter based on co-occurrence statistics (total number of co-occurrences of a given pair is calculated by considering the total number of co-occurrences across all sentences in all documents) and an extended version of Normalized Point-wise Mutual Information (NPMI) [19] association strength measurement to reduce noise possibly introduced by the high recall, low precision co-occurrence method. We selected the associations (between pathogen and disease classes) having an NMPI value above 0.2 and co-occurring at least 10 times in the literature.

We extended NPMI, which is a measure of collocation between two terms, to a measure of collocation between two classes. Hence, we reformulated the NPMI measure for our application. First, we identify, for every class, the set of labels and synonyms associated with the class (*Labels*(*C*) denotes the set of labels and synonyms of *C*). We then define *Terms*(*C*) as the set of all terms that can be used to refer to *C*: $Terms(C) := \{ x | x \in Labels(S) \land S \sqsubseteq C \}$.

We calculate the NPMI between classes *C* and *D* as

1 $$ npmi(C,D) = \frac{\log{\frac{n_{C,D}\cdot n_{tot}}{n_{C} \cdot n_{D}}}}{-\log{\frac{n_{C,D}}{n_{tot}}}}  $$

where *n*_*tot*_ is the total number of sentences in our corpus in which at least one pathogen and one disease name co-occur (i.e., 4,427,138), *n*_*C*,*D*_ is the number of sentences in which both a term from *Terms*(*C*) and a term from *Terms*(*D*) co-occur, *n*_*C*_ is the number of sentences in which a term from *Terms*(*C*) occurs, and *n*_*D*_ is the number of sentences in which a term from *Terms*(*D*) occurs.

## Results

### Statistics on extracted pathogen–Disease associations

We extracted a total of 3420 distinct pathogen–disease pairs belonging to 316 1357 distinct diseases and pathogens respectively from over 1.8 million Open Access full text articles. To identify the associations, we used a combination of lexical, statistical, and ontology-based rules. We used lexical matches to identify whether the label or synonym of a pathogen or disease is mentioned in a document; we used a statistical measure, the normalized point-wise mutual information, to determine whether pathogen and disease mentions co-occur significantly often in literature; and we used ontologies as background knowledge to expand sets of terms based on ontology-base inheritance.

### Performance evaluation

**Fig. 1:**
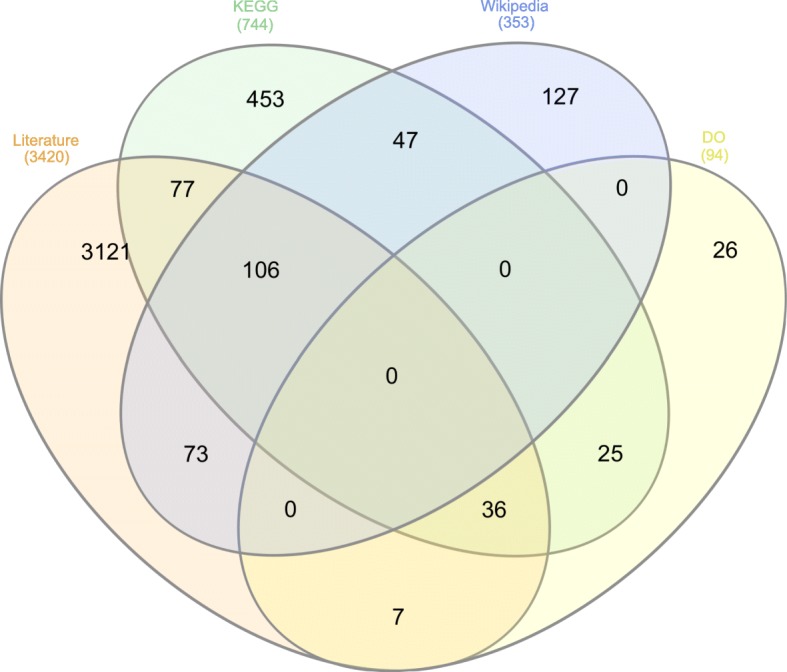
Overlapping pathogen–disease associations between literature and other resources

To evaluate the text mined pathogen–disease associations, we used several manually curated resources including the KEGG [7] database, DO [5], and a list of pathogen–disease associations in Wikipedia [8] as reference, and we compare our results to the information contained in them. We could identify 744 pathogen–disease associations (between 455 distinct pathogens and 331 distinct diseases) in KEGG, 353 pathogen–disease associations in Wikipedia (between 250 distinct pathogens and 245 distinct diseases) and 94 pathogen–disease associations in DO (between 90 distinct pathogens and 41 distinct diseases) for which we could map the pathogen and disease identifiers from NCBI Taxonomy and DO to their identifiers/names in KEGG, DO and Wikipedia. Figure 1 shows the overlapping and distinctly identified pathogen–disease associations from these resources and literature.

The recall of our method is 29.4% (219) for KEGG, 50.7% (179) for Wikipedia, 45.7% (43) for DO. There are 525 pairs in KEGG, 174 pairs in Wikipedia and 51 pairs in DO which we could not cover by text mining. The main reason we cannot identify an association is due to limitations in our named entity and normalization procedure as well as its non-existence in the literature.

In addition to the information contained in existing databases, we extracted many more associations from literature (3121 in total). To determine the accuracy of these associations, first we randomly selected 50 pathogen–disease pairs and all of the evidence sentences linked to them. We applied our threshold values based on NPMI and number of co-occurrences to distinguish between positive and negative associations; we then manually analyzed the evidence sentences linked to these associations (each association are extracted from one or more sentences) to classify each positive association as either False Positive or True Positive and each negative association either as True Negative or False Negative (manual evaluation data is freely available [20]).

In our manual evaluation, we achieve a precision of 64%, a recall of 89% and an F-score of 74%. The false positives were mainly due to ambiguous abbreviations and pathogen names. For example, “Katanga” which is a geographical place name was annotated as a pathogen name (NCBITaxon:966285) by our method.

Some false negatives were due to rejections by the system based on the threshold settings. For example, “Bartonellosis” (DOID:11102) and “Bartonella ancashensis” (NCBITaxon:1318743) which is also covered by KEGG co-occurred only two times (in two different articles, PMCID:4102455 and PMCID:5382735) in our corpus and therefore the association between them was rejected as we limited our analysis to pathogen–disease pairs that co-occurred ten or more times. Other false negatives were due to missing pathogen or disease labels in our dictionaries. For example, our system could not identify a KEGG covered association between “necrotizing ulcerative gingivitis” (DOID:13924) and “Fusobacterium nucleatum” (NCBITaxon:851) since we included only the infections disease branch of DO in our disease dictionary while “necrotizing ulcerative gingivitis” is not a sub-class of “infectious disease” in DO.

## Discussion

By using ontologies as background knowledge to expand our sets of terms and labels, it is possible to identify pathogen–disease associations even if the labels and synonyms directly associated with the pathogen or disease are not directly found to co-occur in text. For example, we extracted a total of 44 distinct pathogen–disease associations relevant to *dengue disease* (DOID:11205). Twelve our of 44 associations are the direct associations of *dengue disease* (i.e., a label or synonym of the disease is explicitly mentioned in text) while the remaining 32 are indirect associations obtained from associations with labels and synonyms of the sub-classes *asymptomatic dengue* (DOID:0050143), *dengue hemorrhagic fever* (DOID:12206), and *dengue shock syndrome* (DOID:0050125). In total, we found 812 pathogen–disease associations which do not directly co-occur in literature but are inferred through the ontology.

The performance of our system depends on two parameters: the NPMI value and the number of co-occurrences used as a threshold. In the future, we may use these two values to automatically determine optimal threshold based on a more comprehensive evaluation set of pathogen–disease associations which needs to be created and could also be useful for developing machine learning based methods. While our initial text mining approach performs at a promising level (F-score 74%), there is still some room for improvements. As we found the pathogen names to be ambiguous with other domain specific names, we plan to further improve the abbreviation and name filters we apply. For improving the recall of our system, it may be possible to expand our dictionaries with other resources covering disease and pathogen names such as the Experimental Factor Ontology (EFO) [21] and the Unified Medical Language System (UMLS) [22] for diseases, and the Encyclopedia of Life [23] for pathogens.

## Conclusion

Here, we present a text mining method for extracting pathogen–disease associations from the biomedical literature. Our method performed at a promising level with some room for improvements. In future, we plan to improve our text mining method by developing and integrating a pathogen abbreviation filter and expanding the coverage of our pathogen and disease dictionaries. In the scope of infectious disease research, we have included our results in a database of pathogens and the phenotypes they elicit in humans. We believe that our results can further support infectious disease research.

## Abbreviations

DO: Human disease ontology
EFO: Experimental factor ontology
KEGG: Kyoto encyclopedia of genes and genomes
NPMI: Normalized point-wise mutual information
UMLS: Unified medical language system
WHO: World health organisation
